# BIKE: Dietary Exposure Model for Foodborne Microbiological and Chemical Hazards

**DOI:** 10.3390/foods10112520

**Published:** 2021-10-20

**Authors:** Jukka Ranta, Antti Mikkelä, Johanna Suomi, Pirkko Tuominen

**Affiliations:** Risk Assessment Unit, Finnish Food Authority, 00790 Helsinki, Finland; antti.mikkela@foodauthority.fi (A.M.); johanna.suomi@foodauthority.fi (J.S.); pirkko.tuominen@foodauthority.fi (P.T.)

**Keywords:** exposure assessment, Bayesian, model, uncertainty, variability, foodborne hazard, dietary, microbiological, chemical

## Abstract

BIKE is a Bayesian dietary exposure assessment model for microbiological and chemical hazards. A graphical user interface was developed for running the model and inspecting the results. It is based on connected Bayesian hierarchical models, utilizing OpenBUGS and R in tandem. According to occurrence and consumption data given as inputs, a specific BUGS code is automatically written for running the Bayesian model in the background. The user interface is based on shiny app. Chronic and acute exposures are estimated for chemical and microbiological hazards, respectively. Uncertainty and variability in exposures are visualized, and a few optional model structures can be used. Simulated synthetic data are provided with BIKE for an example, resembling real occurrence and consumption data. BIKE is open source and available from github.

## 1. Introduction

Exposure assessment is one of the four parts in risk assessments, the other parts being hazard identification, hazard characterization and risk characterization. Here, we focus on exposure assessment. Foodborne exposure assessment relies on both occurrence data and food consumption data. While the former provides information on the prevalence and level of contamination in foods or food ingredients, the latter provides information on how often and in what amounts the foods are consumed. From this knowledge, estimates of both short-term (‘acute’) and long-term (‘chronic’) mean exposures can be drawn. However, significant uncertainties prevail since the data may be very unbalanced and limited. Probabilistic simulation models can be used to describe both variability and uncertainty, but appropriate statistical *inference* is required for an evidence based approach. Previous non-Bayesian methods have been discussed in e.g., [1,2]. While parametric models [3] and non-parametric empirical Monte Carlo distributions, or both [4,5] have been implemented in other approaches, the uncertainties are seldom modelled in fully probabilistic framework with Bayesian inference for all uncertain quantities jointly. For non-Bayesian approaches it is common that parameter estimation is broken into separate steps with unrelated estimation methods and possibly combined with assumptions for ‘nuisance parameters’ that are part of the model but not the primary target of inference. All this may lead to incoherent quantification of the truly multidimensional uncertainty. The methods are often joined by bootstrapped uncertainty, which can be problematic with small data. The combination of separated estimations does not support a coherent multidimensional uncertainty quantification of the full set of parameters in a holistic manner. For Bayesian approaches, albeit theoretically consistent, the specific obstacles are still the computationally demanding MCMC algorithms and issues with their convergence [6]. Also, insufficient methodological training for risk assessors prevails. Parametric and non-parametric approaches were discussed in [7,8], but examples of Bayesian models are less extensively described. Open-source codes are not commonly available with the existing approaches, preventing detailed comparisons, model development and modifications by others. Hence, the full potential of multidimensional Bayesian hierarchical modeling in open-source format remains underexplored and the Bayesian methods are still often only mentioned as a possible refined approach [7,9], and left to be explored in further studies.

In food risk assessments, both chemical and microbiological hazards need to be assessed accounting for their similarities and differences in statistical analysis. Yet, the existing methods predominantly focus on one or the other separately [10]. There is need to assess several hazards, microbiological and chemical, at the same time for efficient use of risk assessment resources. Separate assessments based on different models and estimation methods for each hazard also compromise the comparability of the resulting estimates when, by large, they could be based on the same cross cutting model. Ultimately, exposures from both should be compared towards a combined risk estimation and risk ranking, which is essential in burden of disease assessments and prioritization.

Here we present BIKE, a model for foodborne exposure assessment, based on Bayesian statistical methods. Foodborne exposure assessment is a crucial part of risk assessments on food safety and nutrition. Generally, foodborne hazards occur in several food types or their ingredients, making it necessary to assess total exposure from multiple food groups based on the food consumption in the studied population group and on the occurrence and concentration levels of the studied hazards in the foods in question. BIKE differs from the existing exposure assessment models in two significant ways. Firstly, it is to our knowledge the only tool combining both chemical (including nutritional) and microbiological exposure assessment, whereas the more widely used models like MCRA [4] are developed only for chemical hazards. Secondly, the use of Bayesian methods in BIKE allow fully probabilistic and evidence based quantification of uncertainty. Both variability and uncertainty are described by probability distributions.

The method in BIKE provides a very general approach for probabilistic inference from all data to all unknown parameters that can be extended to larger models exploiting Bayesian evidence synthesis from several data sources, also with fairly scarce data. The challenges are in implementing the computations. Some Bayesian dietary exposure estimation models have been previously developed and tailored to particular applications and data types [11,12,13,14,15]. However, no general open source Bayesian model has been available. The aim of BIKE is to provide a modifiable Bayesian probabilistic inference method for the exposure assessment of both microbiological and chemical contaminants, suitable for analysing available data. The first user interface was developed to run some basic models without programming skills, requiring only the data in a tabular format from which the Bayesian model is automatically built by BIKE. Additionally, the resulting code for Bayesian models can be freely viewed, extended and used independently by anyone with some experience of BUGS model definitions [16] and R software [17]. Hence, the first model(s) can be used as a starting point for further modeling.

In the next sections, the method is summarized, and the inference models for chemical and microbiological occurrence data are introduced. In the following sections, the inference models for consumption data are described in detail. After these, the Bayesian exposure assessment implied by the Bayesian inference is explained likewise. In the final sections the options in the user interface of BIKE (a shiny app) are briefly demonstrated, concluding in a discussion of methodological aspects. Artificial data were generated as described in Appendix A to provide templates for the data format and as a try-out data.

## 2. Materials and Methods

### Quantifying Uncertainty with Probability: Theory in Short

As a method for evidence based research, Bayesian theory has a simple unifying principle for quantitative inference problems: to estimate unknown parameter(s) θ (or other unknown quantities) from data one starts with a joint distribution of both θ and data variables. This is constructed from the conditional distribution for data variables, given θ (i.e., “the model of data”), and the initial uncertainty (i.e., prior distribution) for parameter(s) θ of the model. Then the prior uncertainty is to be updated to a new uncertainty (i.e., posterior distribution) for θ, given the now observed data values. Bayesian statistical inference [16,18,19] is thus simultaneously applied to all unknown parameters or variables, conditionally on the known ones. The ’known’ is the evidence base consisting of all observed data or known facts. For dietary exposure assessment, such base consists of occurrence and consumption data, while the unknown parameters represent e.g., means and variances of concentration and consumption distributions. For estimating parameter(s) θ from specified data, the posterior probability density function is formally solved as f(θ∣data)=f(data∣θ)f(θ)/C where *C* is a normalizing constant, f(θ) is the prior distribution of the unknown(s), and f(data∣θ) is the probability distribution for all observable variables in data that must depend on the unknown parameter(s) in question. When all variables have been set according to data, the latter is also called likelihood function L(θ)=f(data∣θ) for the unknown parameter(s) θ and it contains all the evidence (external to the prior) for the parameter(s), that can be drawn from the now fixed data. Hence, apart from the constant term, posterior distribution of unknown parameter(s) θ is always proportional to the product form

f(θ∣data)∝L(θ)×f(θ)

where *L* must contain probability models for full data, just like f(θ) must define a prior distribution for all the unknown parameters. This equation is also known as Bayes theorem. Several conditionally independent data sources and observations can then be combined into a full likelihood by multiplication of the likelihood components corresponding to each part of data. For example,

$$\prod_{i=1}^{n}L_{i}\left(\theta \right)=\prod_{i=1}^{n}f\left({\mathrm{data}}_{i}|\theta \right)$$

for each of the *n* data sets. Then, posterior distribution can be written, apart from a normalizing constant, proportional to

$$f\left(\theta |{\mathrm{data}}_{1},\ldots ,{\mathrm{data}}_{n}\right)\propto \prod_{i=1}^{n}L_{i}\left(\theta \right)f\left(\theta \right).$$

This modular structure extends to large hierarchical Bayesian models and is also the backbone of the BIKE models. The essential idea is thus to ’divide and conquer’ by including each partial data with a suitable model, providing us with the likelihood contributions $L_{i}$, and to combine them with the prior f(θ). In general, when these elements are defined over all data, the computation can proceed as numerical simulation from the full posterior distribution using e.g., OpenBUGS [20], JAGS [21] or Stan [22], without having to solve the (difficult) normalizing constant, nor the whole posterior distribution, algebraically. Effectively, this allows putting more focus on building the models for occurrence and consumption instead of dwelling on computational issues of specific sampling techniques, although those cannot be always completely avoided.

This methodological principle is unfolded in detail over the sections below, describing Bayesian inference from the hazard occurrence data and food consumption data, with two main options therein. Each part of data provides a contribution $L_{i}$ to the equation of full posterior distribution as above. Hence, the data will determine what components $L_{i}$ are included in a model. Finally, exposure estimation is the result from the Bayesian inference model by combining probabilistically the hazard occurrence distributions with consumption distributions, which depend on the unknown parameters inferred from data.

## 3. Bayesian Inference from Chemical and Microbiological Occurrence Data

Occurrence data for both chemical and microbiological hazards in a food or food ingredient (we refer to both as ‘food types’) are often drawn from reports of measured concentration values. Some values could be statistically interval censored [23] between limit of quantification (LOQ) and limit of detection (LOD), and some might be left-censored below LOD [24,25,26]. Both chemical and microbiological data should report LOD and LOQ limits. Concentrations of chemical hazards are typically reported in micrograms or milligrams per gram or kilogram. Concentrations of microbiological hazards are assumed to follow the typical format of reported colony forming units (CFU) per mass or volume, e.g., “2 CFU/g”. These type of data are still most often found in reports and other published data sources [27,28,29,30,31]. However, more accurate microbiological data would represent actual plate counts, e.g., “50 CFU in a 25 g analytical sample”, instead of the less informative “2 CFU/g”. Likewise, more accurate data could report patterns of dilution series [32] with information on the number of tubes and the sample volumes and dilutions used. Such detailed data are rarely reported and models exploiting them could be implemented in further versions of BIKE. The use of likelihood based methods for estimating concentration distributions has been advocated over simple substitution methods [33,34], although the proportion of censored values should not be extremely high. It is necessary to require at least some exact values above LOQ to be able to estimate the concentration distribution at all [35]. The amount of data is directly reflected in the uncertainty of the parameter estimates for the distribution. The default distribution in BIKE is log-normal. Evidence for log-normality as a general feature has been proposed for pesticide concentrations [36]. A convenient feature is also that the product of log-normal variables is also log-normal. This is useful since exposure is a product of two random variables: concentration and consumption. Furthermore, hierarchical structures with variance components for describing nested variability between groups, individuals, measurements, etc. are based on normal distributions for transformed (e.g., log) variables. Similar model structures and Bayesian inference have been previously applied to observed intakes accounting for a mixture population of high and low consumers [14] with observed seven days per consumer. The total likelihood contribution that needs to be constructed from occurrence data will depend on whether the data contain only exact values, or additionally some censored values. The left-censored data can be interpreted in two ways: either they all represent non-zero positive values, or part of them could be truly zeros. Both interpretations are options in BIKE for the user to indicate when specifying the data. This information is then read from the data by BIKE, and it implies two different models, described in the next sections.

### 3.1. Option 1: Distribution of Positive Concentrations and Contamination Prevalence Estimated Separately

The first option is to assume that all reported measurements of concentration values $c_{ijk}$, even if below LOD, represent truly positives, with $f(\log\left(c_{ijk}\right)|,\mu_{ij}^{h},\sigma_{ij}^{h})$ as a probability density function of normal distribution with mean $\mu_{ij}^{h}$ and standard deviation $\sigma_{ij}^{h}$, and *F* as the corresponding cumulative probability function per hazard *i* in food type *j*. The possible likelihood contributions for the Bayesian model are either from $n_{1}$ exact values $c_{ijk}$, or some censored values

$$\begin{array}{cc} L_{1}=\prod_{k=1}^{n_{1}}f\left(\log\left(c_{ijk}\right)|\mu_{ij}^{h},\sigma_{ij}^{h}\right) & \mathrm{if}\;c_{ijk}>{\mathrm{LOQ}}_{ijk}\;,\;\mathrm{exact}\\ L_{2}=\prod_{k=1}^{n_{2}}F\left(\log\left({\mathrm{LOQ}}_{ijk}\right)|\mu_{ij}^{h},\sigma_{ij}^{h}\right)-F\left(\log\left({\mathrm{LOD}}_{ijk}\right)|\mu_{ij}^{h},\sigma_{ij}^{h}\right) & \mathrm{if}\;{\mathrm{LOD}}_{ijk}<c_{ijk}<{\mathrm{LOQ}}_{ijk}\\ L_{3}=\prod_{k=1}^{n_{3}}F\left(\log\left({\mathrm{LOD}}_{ijk}\right)|\mu_{ij}^{h},\sigma_{ij}^{h}\right) & \mathrm{if}\;c_{ijk}<{\mathrm{LOD}}_{ijk} \end{array}$$

so that one or several of these will apply according to which of them correspond to data instances. For a meaningful estimation, at least some measurements need to be exact values. Since $L_{1},L_{2},L_{3}$ are applicable to the estimation of distribution parameters of strictly positive concentrations, additional information in data is required for inferring the prevalence of positives. Sample data with sample size and sample positives provide this additional binomial likelihood contribution

$$L_{4}=\mathrm{Binomial}\left(x_{ij}|q_{ij},n_{ij}\right).$$

The posterior distribution of model parameters is then constructed from one of the possible evidence combinations

$$\begin{array}{ccc} & f(\mu_{ij}^{h},\sigma_{ij}^{h},q_{ij}|\mathrm{evidence}\;1,4) & \propto L_{1}L_{4}f\left(\mu_{ij}^{h},\sigma_{ij}^{h},q_{ij}\right)\\ \mathrm{or} & f(\mu_{ij}^{h},\sigma_{ij}^{h},q_{ij}|\mathrm{evidence}\;1,2,4) & \propto L_{1}L_{2}L_{4}f\left(\mu_{ij}^{h},\sigma_{ij}^{h},q_{ij}\right)\\ \mathrm{or} & f(\mu_{ij}^{h},\sigma_{ij}^{h},q_{ij}|\mathrm{evidence}\;1,3,4) & \propto L_{1}L_{3}L_{4}f\left(\mu_{ij}^{h},\sigma_{ij}^{h},q_{ij}\right)\\ \mathrm{or} & f(\mu_{ij}^{h},\sigma_{ij}^{h},q_{ij}|\mathrm{evidence}\;1,2,3,4) & \propto L_{1}L_{2}L_{3}L_{4}f\left(\mu_{ij}^{h},\sigma_{ij}^{h},q_{ij}\right) \end{array}$$

where $f(\mu_{ij}^{h},\sigma_{ij}^{h},q_{ij})$ is the prior distribution of the parameters. Flat priors are used for $\mu_{ij}^{h},q_{ij}$. For the standard deviation $\sigma_{ij}^{h}$, either uniform distribution is used, or gamma-distribution for inverse variance. If uniform, unrealistically large values are avoided by ad hoc upper limit derived empirically as a multiple of the standard deviation evaluated from observed log-concentrations after adding two extreme values. In further developments, a hierarchical prior might be exploited for combining information from multiple food categories with variations between and within categories.

### 3.2. Option 2: Distribution of Positive Concentrations and Contamination Prevalence Estimated Jointly

In the second option, without having the certainty of which values were true zeros among those below LOD, a zero-inflated distribution is modeled. The likelihood contributions from data are divided into three possible forms as follows. Note that ‘detection’ implies the concentration is over LOD, so that conditional probabilities for concentrations, given detection, obey truncated distributions.

(1) If the observation is below LOD (i.e., not detected):

$$L_{5}=1-P\left(\mathrm{detection}\right)=1-q_{ij}\left(1-F\left({\mathrm{LOD}}_{ijk}\right)\right).$$

(2) If the observation is detected and between LOD and LOQ:

$$L_{6}=P\left({\mathrm{LOD}}_{ijk}<c_{ijk}<{\mathrm{LOQ}}_{ijk}|\mathrm{detection}\right)P\left(\mathrm{detection}\right)$$

$$=F_{{\mathrm{LOD}}_{ijk}}\left({\mathrm{LOQ}}_{ijk}\right)q_{ij}\left(1-F\left({\mathrm{LOD}}_{ijk}\right)\right)=\left[F\left({\mathrm{LOQ}}_{ijk}\right)-F\left({\mathrm{LOD}}_{ijk}\right)\right]q_{ij}$$

where $F_{\mathrm{LOD}\;}$ is the cumulative probability of the truncated distribution over [LOD,∞).

(3) If the observation is detected and over LOQ (thus exact value):

$$L_{7}=P\left(c_{ijk}|c_{ijk}>{\mathrm{LOQ}}_{ijk}\right)P\left(c_{ijk}>{\mathrm{LOQ}}_{ijk}\right)$$

$$=f_{{\mathrm{LOQ}}_{ijk}}\left(c_{ijk}\right)q_{ijk}\left[1-F\left({\mathrm{LOQ}}_{ijk}\right)\right]=\frac{f(c_{ijk})}{1-F({\mathrm{LOQ}}_{ijk})}\left[1-F\left({\mathrm{LOQ}}_{ijk}\right)\right]q_{ijk}=f\left(c_{ijk}\right)q_{ijk}$$

where $f_{\mathrm{LOQ}}$ is the density function of the truncated distribution over [LOQ,∞). In this case, there is no separate model for estimating prevalence $q_{ij}$ because it is already in the expressions $L_{5},L_{6},L_{7}$ jointly with distribution parameters $\mu_{ij}^{h},\sigma_{ij}^{h}$. For a meaningful estimation, at least some measurements need to be exact values. The posterior distribution of model parameters is then one of the following:

$$\begin{array}{ccc} & f(\mu_{ij}^{h},\sigma_{ij}^{h},q_{ij}|\mathrm{evidence}\;7) & \propto L_{7}f\left(\mu_{ij}^{h},\sigma_{ij}^{h},q_{ij}\right)\\ \mathrm{or} & f(\mu_{ij}^{h},\sigma_{ij}^{h},q_{ij}|\mathrm{evidence}\;5,7) & \propto L_{5}L_{7}f\left(\mu_{ij}^{h},\sigma_{ij}^{h},q_{ij}\right)\\ \mathrm{or} & f(\mu_{ij}^{h},\sigma_{ij}^{h},q_{ij}|\mathrm{evidence}\;6,7) & \propto L_{6}L_{7}f\left(\mu_{ij}^{h},\sigma_{ij}^{h},q_{ij}\right)\\ \mathrm{or} & f(\mu_{ij}^{h},\sigma_{ij}^{h},q_{ij}|\mathrm{evidence}\;5,6,7) & \propto L_{5}L_{6}L_{7}f\left(\mu_{ij}^{h},\sigma_{ij}^{h},q_{ij}\right). \end{array}$$

The above construction could also be extended with so-called hidden variables to describe the uncertain true status (binary variable) of each measurement below LOD, but this is not necessary for parametric inference. However, for simulating acute microbiological exposures it will be used for handling true zero occasions as “on/off” variable. The estimation of model parameters $\mu_{ij}^{h},\sigma_{ij}^{h},q_{ij}$ may become very uncertain due to the added uncertainty of concentrations below LOD, which now could have occurred either because they were true zeros (*q* small) or small positive values (μ small). This can even lead to bimodal posterior distributions.

## 4. Bayesian Inference from Consumption Data

Food consumption surveys are costly and time-consuming, and participation in such studies has decreased in the last decades. In addition, surveys are mainly conducted from a nutritional viewpoint and hence some information important for food safety risk assessment may be lost or limited. This calls for flexible probabilistic models for quantitative uncertainty assessment.

With consumption data, the overall assumption in BIKE is that every individual consumes all food types with some positive frequency and some positive amounts in the long run, small or large. In principle, the estimation of absolute non-consumers might be done by e.g., zero-inflated models, but this would require potentially numerous days per individual in food diaries. It is currently more likely that only a very few days are recorded per individual so that a feasible approach is to apply a model without an extra parameter for the proportion of true non-consumers in BIKE. Low observed frequencies then lead to low estimates, which cannot be distinguished from committed absolute zero consumers. However, some other features were included in BIKE model, such as

- between-foods correlation in expected (long-term average) consumption amounts,
- between-foods correlation in one-day consumption amounts,
- between-foods correlation in consumption frequency, and
- either dependency or independency of consumption decisions between days.

### 4.1. Model for Consumption Amounts on Actual Consumption Days

The model for consumption amounts concerns the distribution of daily serving sizes when a food item is consumed, i.e., excluding zero consumption days. The model for consumption data is the same for microbiological and chemical exposure assessment, except that a distribution for long-term mean consumption is estimated for chemical assessment, but a distribution for daily consumption is estimated for microbiological assessment. Also, consumption per body weight is important in chemical assessments whereas consumption in absolute quantity is important in microbiological assessments. Therefore, body weight data are also used for estimating the parameters of a body weight distribution. A log-normal model is applied to the likelihood contribution from the body weights of individuals r=1,…,R

$$L_{w}=\prod_{r=1}f\left(\log\left(w_{r}\right)|\mu_{w},\sigma_{w}\right).$$

Assuming that consumption data are available from (at least) a two-day food diary, the likelihood contribution from observed positive consumption amounts $s_{jrk}$ of food type *j* for an individual consumer respondent *r* over days *k* is modeled as

$$L_{r}=\prod_{k=1}\mathrm{MN}\left(\log\left(s_{1rk},\ldots ,s_{n_{f}rk}\right)|\left(\mu_{1r}^{f},\ldots ,\mu_{n_{f}r}^{f}\right),C_{s}\left[\right]\right)$$

where $\mu_{1r}^{f},\ldots ,\mu_{n_{f}r}^{f}$ is the mean vector for the *r*th consumer in log-scale and $C_{s}\left[\right]$ is the covariance matrix. Note that the correlations do not describe a person’s unique correlations for his/her actual consumed amounts in a day’s meal. To model an individual’s own specific correlations between consumption amounts of food types would require much more observations per individual than are usually available. Note also that zero consumption amounts are technically treated as missing values in the Bayesian estimation since the multinormal distribution describes non-zero positive consumption only. A zero consumption does not provide information on what amount would have been consumed if it had been. The one-day consumption amounts $s_{jrk}$ are expressed as per body weight since this can be directly combined with concentration values to get exposure per body weight for chemical assessments. For microbiological assessments the absolute acute exposure is needed, in which case the predicted $s_{jrk}$ needs to be multiplied by the body weight of the individual. As a possible further development, even regression models could be developed for the consumption, accounting for body weight, age, sex and other factors as explanatory variables. Alternatively, one could stratify the data according to those factors and run the model separately for each stratum.

Expected (average) individual log-consumption amounts $\mu_{jr}^{f}$ can also be correlated between food types $f=1,\ldots ,n_{f}$ so that, in general, some food types tend to be more (or less) consumed in combinations among the consumers. Hence the model is

$$\left(\mu_{1r}^{f},\ldots ,\mu_{n_{f}r}^{f}\right)∼\mathrm{MN}\left(\left(\mu_{10}^{f},\ldots ,\mu_{n_{f}0}^{f}\right),C_{\mu }\left[\right]\right)$$

where the multivariate normal distribution has population means $\mu_{j0}^{f}$ for each food type *j* and a covariance matrix $C_{\mu }\left[\right]$ describing correlations between mean log-consumption amounts of food types, among all consumers. The prior distribution of $\mu_{0}^{f}$ is a vague normal distribution, and the priors for the inverse of covariance matrices $C_{s}\left[\right]$ and $C_{\mu }\left[\right]$ are Wishart distributions. The mean consumption amounts $E\left(s_{jrk}\right)=\exp\left(\mu_{jr}^{f}+0.5C_{s}\left[j,j\right]\right)$ for individuals are of interest for the assessment of chronic exposure, whereas the random individual consumption amounts $s_{jrk}$ are of interest for the assessment of acute exposure. The model for consumption frequency has two options as selection tabs in the user interface, described in the following sections.

### 4.2. Option 1: Consumption Frequencies Assuming Days Are Independent of Each Other

Consumption surveys attempt to collect a representative random sample of consumers and consumption days [37]. Because the day count per consumer often cannot be larger than two, one typically wants to avoid situations where both days would represent exceptional periods, e.g., festive seasons. This is avoided by a survey design with sufficiently long time between two survey days, and randomization. When food diaries are collected over two or more unrelated days, the consumption event (yes/no) on any observation day may be assumed to be independent of the event on a previous observation day. Then, the consumption frequencies $p_{jr}$ are modeled so that the likelihood contribution from consumption ($U_{jrk}=1$) and no-consumption ($U_{jrk}=0$) days follows from Bernoulli-distribution

$$L_{jr}=\prod_{k=1}^{n_{d}}\mathrm{Bernoulli}\left(U_{jrk}|p_{jr}\right)$$

Although the $U_{jrk}$ are independent given $p_{jr}$, the consumers’ logit-frequencies in a population group can be correlated between food types $f=1,\ldots ,n_{f}$ so that

$$\left(\mathrm{logit}\left(p_{1r}\right),\ldots ,\mathrm{logit}\left(p_{n_{f}r}\right)\right)∼\mathrm{MN}\left(\left(\mathrm{logit}\left(p_{10}\right),\ldots ,\mathrm{logit}\left(p_{n_{f}0}\right)\right),C_{p}\left[\right]\right)$$

where the multivariate normal distribution has population means $\mathrm{logit}(p_{j0})$ for each food type *j* and a covariance matrix $C_{p}\left[\right]$ describing correlations between consumption frequencies of food types. The consumption frequencies of individual consumers $p_{jr}$ are of interest for chronic exposure assessment, whereas the random individual consumption events $U_{jrk}$ (‘on/off’) are of interest for acute exposure assessment.

### 4.3. Option 2: Consumption Frequencies Assuming the Consumption on a Day Depends on the Previous Day

If consumption survey is based on consecutive days (e.g., 48 h recalls), the consumption events may be correlated over days. This means, if a food item is consumed today, it may be more (or less) likely consumed also tomorrow. The consecutive days are not independent then. Moreover, for microbiological exposures the bacterial growth is particularly important when the consumption of the same food item continues over a few days. Such information can only be obtained when two or more consecutive days are reported in dietary survey data. Even then, it is usually not recorded whether exactly the same food package is kept overnight and consumed repeatedly, although it might be likely so. With such assumption, the simplest model is a Markov chain with transition probabilities for daily consumption events. The probability depends on whether the consumption occurred on previous day. This is concisely expressed as a transition probability matrix $P_{j}$, for food *j*:

$$P_{j}=\left\{\begin{array}{ccc} p_{j}^{01} & = & P(\mathrm{consumes}\;\mathrm{food}\;j|\mathrm{did}\;\mathrm{not}\;\mathrm{consume}\;\mathrm{food}\;j\;\mathrm{previous}\;\mathrm{day})\\ p_{j}^{11} & = & P(\mathrm{consumes}\;\mathrm{food}\;j|\mathrm{consumed}\;\mathrm{food}\;j\;\mathrm{previous}\;\mathrm{day})\\ p_{j}^{00} & = & 1-p_{j}^{01}\\ p_{j}^{10} & = & 1-p_{j}^{11} \end{array}\right.$$

From this Markov chain it follows that the long run (stationary) probability of consuming the food on an arbitrary day can be written as $p_{j}=p_{j}^{01}/\left(p_{j}^{01}+p_{j}^{10}\right)$. For a more detailed exposure assessment beyond BIKE, this model could be combined with bacteria growth model that gives the initial concentration and the predicted concentrations for each of the following days [38]. The consumption amounts for consumption days are yet modeled as independent of previous day.

In the simple day-to-day model, the two-day (or several days) consumption data provides evidence for the transition probabilities over consecutive days as

$$L_{j}={\left(p_{j}^{01}\right)}^{x_{j}^{01}}{\left(1-p_{j}^{01}\right)}^{n_{0}-x_{j}^{01}}\times {\left(p_{j}^{11}\right)}^{x_{j}^{11}}{\left(1-p_{j}^{11}\right)}^{n_{1}-x_{j}^{11}}$$

where $x_{j}^{01}$$\left(x_{j}^{11}\right)$ is the number of observed transitions 0→1 (1→1) between consecutive days, and $n_{j}^{0}$ ($n_{j}^{1}$) is the number of times when the preceding day was 0 (1). The prior distribution $f(p_{j}^{01},p_{j}^{11})$ is uniform for both parameters. Note that the transition probabilities were not defined for each consumer but as common parameters for the group of consumers. With more observation days per consumer, more personalized transition models might become feasible.

### 4.4. Posterior Distribution for Consumption Model Parameters

All the parameters related to the amounts, frequencies and body weights are summarized as

μwσwμjrfj=1,…,nf,r=1,…,nrCs[]nf×nfμj0fj=1,…,nfCμ[]nf×nfOption1:independentdays:Option2:dependentdays:pjrj=1,…,nf,r=1,…,nrpj01j=1,…,nfpj0j=1,…,nfpj10j=1,…,nfCp[]nf×nf

with the two alternative consumption frequency models as options. The three covariance matrices $C_{s}\left[\right],C_{\mu }\left[\right]$ and $C_{p}\left[\right]$ have usual Wishart priors, which give uniform priors for correlations. The expressions for full posterior distributions are constructed by multiplying the prior distributions with the likelihood expressions $L_{w},L_{r},L_{jr},L_{j}$ according to the data and model option.

## 5. Exposure Assessment Implied by Bayesian Inference

After the posterior distribution for all model parameters is simulated, the exposure assessments follow directly from it. An exposure distribution either describes the variation in random daily acute exposures among all consumers, or the variation in long-term mean exposures among all consumers. Every exposure distribution can be viewed as a variability distribution that depends on its uncertain parameters that were the objects of inference in the Bayesian models described above. Hence, each set of parameter values corresponds to one possible exposure distribution. In simple cases, the exposure distribution is solved as a log-normal distribution with specific expression of parameters, but otherwise it is simulated based on the parameters of the underlying distributions. There are several possible ways to summarise exposures in BIKE, and these are available as selection tabs for the user to choose in the results.

### 5.1. Univariate (Marginal) Acute Positive Exposure for Single Food Type, Single Microbiological Hazard

This is a variability distribution of acute microbiological expected doses (consumption × concentration) among consumers, for all the *consumption days* of a specific *contaminated* food type per specific microbiological hazard. Since this variability distribution (log-normal) depends on uncertain parameters, several possible variability distributions are overlaid in the plots to visualize uncertainty. Thus, uncertainty is simulated for the log-normal variability distribution. The frequency of consuming such contaminated food (exposure frequency) is an uncertain quantity for which estimates are presented numerically.

### 5.2. Univariate (Marginal) Chronic Positive Exposure for Single Food Type, Single Chemical Hazard

This is a variability distribution of chronic chemical exposures among consumers, for the *consumption days* of a specific *contaminated* food type, per specific chemical hazard. Since this variability distribution (log-normal) depends on uncertain parameters, several possible variability distributions are overlaid in the plots to visualize uncertainty. Thus, uncertainty is simulated for the log-normal variability distribution. The frequency of consuming such contaminated food (exposure frequency) is an uncertain quantity for which estimates are presented numerically.

### 5.3. Multiple Exposure from a Subset of Selected Foods among All Food Types, Single Hazard

This variability distribution does not have analytical solution and hence it needs to be simulated for both chemical (chronic) and microbiological (acute) assessment. Expressing uncertainty about the variability distribution, e.g., its cumulative distribution function and its quantile points, requires 2D simulation of variability and uncertainty [39]. Simulations can account for consumption and contamination frequencies for all days, or simulations can represent only actual consumption days and only non-zero positive contamination of the foods. For microbiological hazards, a Poisson distribution provides the final random dose variability in servings. A bacteria dose may become zero even from a contaminated food if the concentration is low and/or consumed amount is small, due to randomly scattered bacteria cells.

### 5.4. Posterior Predictive Distributions

Posterior predictive distributions for acute microbiological or chronic chemical exposures can be simulated. Likewise, for the consumption of foods and the concentrations of hazards in foods. These distributions integrate both variability and uncertainty into an overall assessment of total uncertainty (both aleatory and epistemic uncertainty). In other words, posterior predictive distribution is a weighted average of all possible variability distributions. It is weighted with respect to the posterior distribution of the uncertain parameters when each parameter set defines a variability distribution. In the limit, if the amount of data (sample size) grows, the posterior distribution of parameters becomes more peaked around the true parameter values, and hence the predictive distribution approaches the true variability distribution, assuming it exists among the parametric family of distributions in question.

### 5.5. Microbiological Acute Exposures

Microbiological exposure may lead to acute infection and illness from a single serving. Therefore, long run mean exposures are not very meaningful whereas the distribution of acute exposures is required for risk assessment and is provided in BIKE.

#### 5.5.1. Exposure to a Hazard-Food Pair

As a default, log-normal distributions are used for modeling the variation in individual positive daily consumption amounts and in the individual mean consumption amounts for those consumption days. Hence, the positive (absolute) acute consumption amount of a single food *j* for a random consumer *r* has univariate distribution that is log-normal

$$s_{jr}w_{r}∼\mathrm{LN}\left(\mu_{0j}^{f}+\mu_{w},C_{s}\left[j,j\right]+C_{\mu }\left[j,j\right]+\sigma_{w}^{2}\right)$$

with parameters $\mu_{0j}^{f}+\mu_{w}$ and $C_{s}\left[j,j\right]+C_{\mu }\left[j,j\right]+\sigma_{w}^{2}$ due to hierarchical normal model for the logarithms with two variance components, $C_{s}\;\&\;C_{\mu }$, and assuming log-normal body weight is independent of consumption in the studied consumer group. Note that the consumption model parameters are estimated from $s_{jr}$ as per body weight, and hence a multiplication by body weight $w_{r}$ is needed for simulating absolute amounts.

Log-normal distributions are also the default for the variation of concentrations, which are independent of consumption amounts. Hence, the resulting acute exposure to a contaminated food type *j* has again a univariate log-normal distribution. For each hazard-food combination, the univariate distribution of acute microbiological exposures, due to consuming contaminated food *j* is:

$$e_{ijr}^{+}=s_{jr}w_{r}c_{ij}∼\mathrm{LN}(\mu_{0j}^{f}+\mu_{ij}^{h}+\mu_{w},C_{s}\left[j,j\right]+C_{\mu }\left[j,j\right]+{\left(\sigma_{ij}^{h}\right)}^{2}+\sigma_{w}^{2}).$$

Uncertainty distribution for the mean E() and median $Q_{50\%}\left(\right)$ of acute exposure due to consuming contaminated food *j* is obtained by plotting the posterior distributions of the expressions:

(1) $$E\left(e_{ijr}^{+}\right)=\exp\left(\mu_{0j}^{f}+\mu_{ij}^{h}+\mu_{w}+0.5C_{s}\left[j,j\right]+0.5C_{\mu }\left[j,j\right]+0.5{\left(\sigma_{ij}^{h}\right)}^{2}+0.5\sigma_{w}^{2}\right)$$

and

(2) $$Q_{50\%}\left(e_{ijr}^{+}\right)=\exp\left(\mu_{0j}^{f}+\mu_{ij}^{h}+\mu_{w}\right).$$

For microbiological hazards, the uncertainty of the univariate distribution for $e_{ijr}^{+}$ is provided in BIKE by showing a few possible distribution functions, each corresponding to randomly drawn (uncertain) parameters from the posterior distribution. The estimated exposure frequency, i.e., the proportion $p_{jr}q_{ij}$ of days when the consumption of a contaminated food occurs is given numerically within graphics.

#### 5.5.2. Exposure to Several Food Types Summed up

The distribution for the summed acute exposure $\sum_{j}e_{ijr}^{+}$ due to consuming many contaminated food types does not have a standard algebraic solution. The exposure distributions, which include possible zero consumption days and zero contamination, for acute exposures $e_{ijr}$ to a food type *j* or all food types $e_{ir}$, do not either have solutions as standard distributions. However, given the core parameters, the distributions for summed acute exposures can be produced by sampling in a sequence from the conditional distributions:

$$\begin{array}{cc} \mathrm{logit}\left(p_{1r}\right),\ldots ,\mathrm{logit}\left(p_{n_{f}r}\right) & ∼\mathrm{MN}\left(\mathrm{logit}\left(p_{0}\right),C_{p}\left[\right]\right)\;\;\mathrm{or}\;p_{jr}=p_{j}^{01}/\left(p_{j}^{01}+p_{j}^{10}\right)\\ U_{jr} & ∼\mathrm{Bernoulli}(p_{jr})\\ \mu_{1r}^{f},\ldots ,\mu_{n_{f}r}^{f} & ∼\mathrm{MN}(\mu_{0}^{f},C_{\mu }\left[\right])\\ \log\left(s_{1r}\right),\ldots ,\log\left(s_{n_{f}r}\right) & ∼\mathrm{MN}(\mu_{r}^{f},C_{s}\left[\right])\\ w_{r} & ∼\mathrm{LN}(\mu_{w},\sigma_{w}^{2})\\ c_{ij} & ∼\mathrm{LN}(\mu_{ij}^{h},{\left(\sigma_{ij}^{h}\right)}^{2})\\ e_{ijr}^{+} & =s_{jr}c_{ij}w_{r}\\ I_{ijr} & =\mathrm{Bernoulli}(q_{ij})\\ e_{ijr} & =I_{ijr}U_{jr}e_{ijr}^{+}\\ e_{ir} & =\sum_{j=1}^{n_{f}}e_{ijr} \end{array}$$

Hence, the distributions for acute exposures $e_{ir}$ are most efficiently produced separately in R after the parameter values sampled from the posterior distribution have been saved from each MCMC iteration. Then also the uncertainty about the variability distribution can be quantified by 2D simulation, or the more straightforward posterior predictive distribution simulated. For microbiological hazards, chronic exposure is not usually relevant and is (currently) not provided in BIKE results.

### 5.6. Chemical Chronic Exposures

Unless a chemical can cause health effects due to acute exposure, the occasional extreme exposures are not of concern as high and low values are averaged in the long run exposure. Therefore, only chronic exposure to chemicals is estimated in BIKE. This requires modeling the population distribution of individual mean exposures.

#### 5.6.1. Exposure to a Hazard-Food Pair

Using the two marginal univariate hierarchical log-normal models

$$s_{jr}∼\mathrm{LN}\left(\mu_{jr}^{f},C_{s}\left[j,j\right]\right)\;\;\;,\;\;\;\exp\left(\mu_{jr}^{f}\right)∼\mathrm{LN}\left(\mu_{0j}^{f},C_{\mu }\left[j,j\right]\right)$$

the chronic long term (mean) consumption amount due to daily consumption of food *j* of a random consumer *r* is

$$E\left(s_{jr}\right)=\exp\left(\mu_{jr}^{f}+0.5C_{s}\left[j,j\right]\right)=\exp\left(\mu_{jr}^{f}\right)\exp\left(0.5C_{s}\left[j,j\right]\right)∼\mathrm{LN}\left(\mu_{0j}^{f}+0.5C_{s}\left[j,j\right],C_{\mu }\left[j,j\right]\right)$$

In the assessment of chronic exposure, variation in hazard concentrations is replaced by the expected (mean) concentration, so that the variability in chronic exposure is only due to variable mean consumption between individuals. The between consumer variation in chronic exposures from daily consumed contaminated food *j* has univariate distribution

$$E\left(e_{ijr}^{+}\right)=E\left(s_{jr}\right)E\left(c_{ij}\right)∼\mathrm{LN}\left(\mu_{0j}^{f}+0.5C_{s}\left[j,j\right]+\log\left(E\left(c_{ij}\right)\right),C_{\mu }\left[j,j\right]\right)$$

where the mean concentration is $E\left(c_{ij}\right)=\exp\left(\mu_{ij}^{h}+0.5{\left(\sigma_{ij}^{h}\right)}^{2}\right)$. Uncertainty distribution for the mean and median chronic exposure from consumption days is obtained by plotting the posterior distributions of the expressions:

(3) $$E\left(E\left(e_{ijr}^{+}\right)\right)=\exp\left(\mu_{0j}^{f}+0.5C_{s}\left[j,j\right]+\log\left(E\left(c_{ij}\right)\right)+0.5C_{\mu }\left[j,j\right]\right)$$

and

(4) $$Q_{50\%}\left(E\left(e_{ijr}^{+}\right)\right)=\exp\left(\mu_{0j}^{f}+0.5C_{s}\left[j,j\right]+\log\left(E\left(c_{ij}\right)\right)\right).$$

The uncertainty of the univariate distribution for $E(e_{ijr}^{+})$ is provided in BIKE by showing a few possible distribution functions, each corresponding to randomly drawn parameters from the posterior distribution. The estimated exposure frequency $p_{jr}q_{ij}$ is given numerically within graphics. Chronic exposure, including possible zero exposures of food type *j*, for an individual *r* is

$$E\left(e_{ijr}\right)=q_{ij}p_{jr}E\left(e_{ijr}^{+}\right)$$

This has variability distribution between individuals arising from the product of two consumer specific random variables $p_{jr}$ and $E(e_{ijr}^{+})$. The marginal distribution of $E(e_{ijr}^{+})$ is log-normal, and the marginal distribution of $p_{jr}$ is logit-normal. Alternatively, for a consumer group: $p_{jr}=p_{j}^{01}/\left(p_{j}^{01}+p_{j}^{10}\right)$ if the Markov chain model is used for consumption frequency. In either case, the variability distribution of $E(e_{ijr})$ needs to be simulated from the conditional distributions for individuals.

#### 5.6.2. Exposure to Several Food Types

Variation of individual chronic exposure due to many food types $E(e_{ir})$ including proportion of zero exposures, needs to be simulated from the conditional distributions

$$\begin{array}{cc} \mathrm{logit}\left(p_{1r}\right),\ldots ,\mathrm{logit}\left(p_{n_{f}r}\right) & ∼\mathrm{MN}\left(\mathrm{logit}\left(p_{0}\right),C_{p}\left[\right]\right)\;\;\mathrm{or}\;p_{jr}=p_{j}^{01}/\left(p_{j}^{01}+p_{j}^{10}\right)\\ \mu_{1r}^{f},\ldots ,\mu_{n_{f}r}^{f} & ∼\mathrm{MN}(\mu_{0}^{f},C_{\mu }\left[\right])\\ E(e_{ijr}^{+}) & =E\left(s_{jr}\right)E\left(c_{ij}\right)=\exp\left(\mu_{jr}^{f}+0.5C_{s}\left[j,j\right]+\mu_{ij}^{h}+0.5{\left(\sigma_{ij}^{h}\right)}^{2}\right)\\ E(e_{ijr}) & =q_{ij}p_{jr}E\left(e_{ijr}^{+}\right)\\ E(e_{ir}) & =\sum_{j=1}^{n_{f}}E\left(e_{ijr}\right) \end{array}$$

Hence, the distributions for $E(e_{ir})$ can be produced separately in R using the MCMC sample of the parameters, so that the parameter values from one MCMC iteration step provide parameters for one possible variability distribution. Hence the uncertainty about the variability distribution can be quantified by 2D simulation, or the more straightforward posterior predictive distribution can be simulated.

### 5.7. Posterior Predictive Distributions for Acute and Chronic Exposure

#### 5.7.1. Is Separation of Uncertainty and Variability Always Worth It?

Separation of uncertainty and variability generally requires “2D simulations” where the uncertain parameters are first simulated $n_{U}$ times from their uncertainty distribution (i.e., posterior or prior distribution). Then, the quantities representing variability are simulated $n_{V}$ times from the conditional distributions determined by those parameters, for each of the simulated values of the parameters. That calls for $n_{U}\times n_{V}$ simulations, which can become computationally heavy. In contrast, a posterior predictive distribution integrates both uncertainty and variability into a single probability distribution, and is generally produced by simulating only once from the variability distribution per each simulated uncertain parameter, i.e., only $n_{U}$ times. This can be a useful summary of total aleatory & epistemic uncertainty. For example, when quantifying uncertainty separately, the uncertainty distribution of 95% variability quantile exp(μ+1.64σ) of a log-normal variability distribution could be obtained by simulating the two required parameters from their posterior distribution f(μ,σ∣x). If the uncertainty of the parameters is large, the uncertainty of the quantile expression is large too, and higher quantiles would be even more uncertain. The 90% inclusive uncertainty bounds of a 99% quantile may become too wide to be useful and its exact bounds would require huge simulations for taming Monte Carlo error. Nevertheless, the uncertainty about the quantile is not uniform over such wide range. The variability quantile lies more probably within the lower side of the uncertainty interval than in the upper. Posterior predictive distribution of a new unseen observation $x^{*}$ integrates it all into one distribution

$$f\left(x^{*}|x\right)=\int_{0}^{\infty }\int_{-\infty }^{\infty }\underset{\mathrm{variability}}{\underset{︸}{f(x^{*}|\mu ,\sigma )}}\underset{\mathrm{posterior}\;\mathrm{of}\;\mu ,\sigma }{\underset{︸}{f(\mu ,\sigma |x)}}\;d\mu d\sigma =E_{\left(\mu ,\sigma \right)}(f\left(x^{*}|\mu ,\sigma \right)|x)$$

which is what the variability distribution is on average, with respect to the posterior distribution of the uncertain parameters. The exact upper 95% predictive quantile of $f(x^{*}|x)$ is what the 95% quantile of the variability distribution is *expected* to be, considering, not separately, but simultaneously the variability of a quantity and what the uncertain parameters of its distribution might be. This integration generally requires simulation, but not 2D simulation. It is also known that, theoretically, with increasing data sample, the posterior predictive distribution approaches the true variability distribution (assuming the type of distribution itself is correct).

#### 5.7.2. Posterior Predictive Outputs in BIKE

By integrating variability at individual level and variability between individuals, and the uncertainty concerning the parameters of those distributions, the posterior predictive distribution of acute exposure $e_{ir}$ and chronic exposure $E(e_{ir})$ are produced over the MCMC simulation of the full posterior distribution by sampling in a sequence at each iteration step:

- (1) uncertain population parameters $p_{0},C_{p}\left[\right],\mu_{0}^{f},C_{s}\left[\right],C_{\mu }\left[\right],\mu_{ij}^{h},\sigma_{ij}^{h},q_{ij},\mu_{w},\sigma_{w}$,
- (2a) consumers’ variable parameters $p_{jr},\mu_{jr}^{f}$, given the population parameters,
- (2b) consumers’ variable chronic exposures $E(e_{ir})$, given the population parameters (if assessment of chronic exposure),
- (3) consumers’ variable acute exposures $e_{ir}$, given the parameters specific for consumer and population (if assessment of acute exposure),

and plotting the resulting MCMC sample of $E(e_{ir})$ or $e_{ir}$. For microbiological hazards, $e_{ir}$ represents the expected bacteria count according to the concentrations multiplied by consumption amounts per food type and then summed over foods. The final bacteria dose is simulated from Poisson($e_{ir}$) to describe the randomness of the bacteria counts in a serving. Hence, the predictive distribution can produce zero counts even when the food(s) was (were) contaminated, if the contamination level and/or consumption amount was small. Posterior predictive distributions are summarized in a table with 1%, 5%, 10%, 50%, 90%, 95% and 99% posterior predictive quantiles. These quantiles indicate where the quantiles of the corresponding variability distribution are expected to be considering the uncertainty of all underlying parameters.

## 6. Results: Driving BIKE

### 6.1. From Inputs to Bayesian Computations and Results: Shiny App

The workflow of BIKE is simple: once the input data are defined in a correct format (more details in the manual), the modeling and computations in BIKE are automatic. After the simulation runs are completed, the role of the user is to select from the click buttons of available options for various types of results or to re-run simulations with the other model options provided.

#### 6.1.1. Input Data Format

Data for hazard concentrations and the consumption of specific foods needs to be stored in Excel formats, which are then to be converted to text-files beforehand, to be read by BIKE. Synthetic test data are provided for an example of inputs, see Appendix A. The example data are in tabular format in Excel and downloadable from the same source as BIKE. They were randomly generated from distributions that roughly represent real hazards in real foods and real consumption, according to some selected literature, and further processed for the example. These are for demonstration purpose only and as a template for inserting real data in the same format. Since BIKE runs on the user’s local computer, there is no need to upload data over the net, which guarantees data protection.

#### 6.1.2. Automatic Model Construction and Simulation

The actual Bayesian model to be constructed in BIKE is automatically determined from the input data features when reading data. For example, if there are no censored values below LOD/LOQ, then a model for censored data is not needed to be included. This automation corresponds to the possible mathematical models, which depend on what data were seen, as described in the methods section. A BUGS-model code is accordingly written into a file, which is automatically read by OpenBUGS for running the MCMC simulations as a background process. The simulation outputs are then instantly read by R-code for plotting the results in the shiny app. The role of the user is to choose and click from options available in the shiny app interface to create new plots from the once simulated MCMC sample, and these are quick to perform. Some other user options will change the model structure itself or change the simulation length, which will start a new simulation, leading to new results. There is no automatic convergence diagnostics for the MCMC or the MCMC error [6]. Therefore, it is recommended that several MCMC runs of different lengths are tested to check how stable the results are. Some diagnostic plots for model parameters are provided for visual inspection of the simulated output. Particularly, higher quantiles of distributions come with larger uncertainty, which may require much longer simulations than for the uncertainty interval of the median.

#### 6.1.3. Selection of Plots for Results

**Figure 1:**
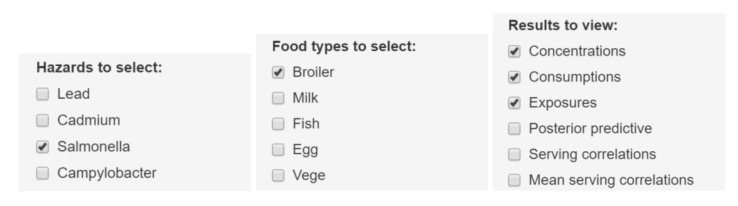
Example of selection tabs for hazards, foods, and results types.

**Figure 2:**
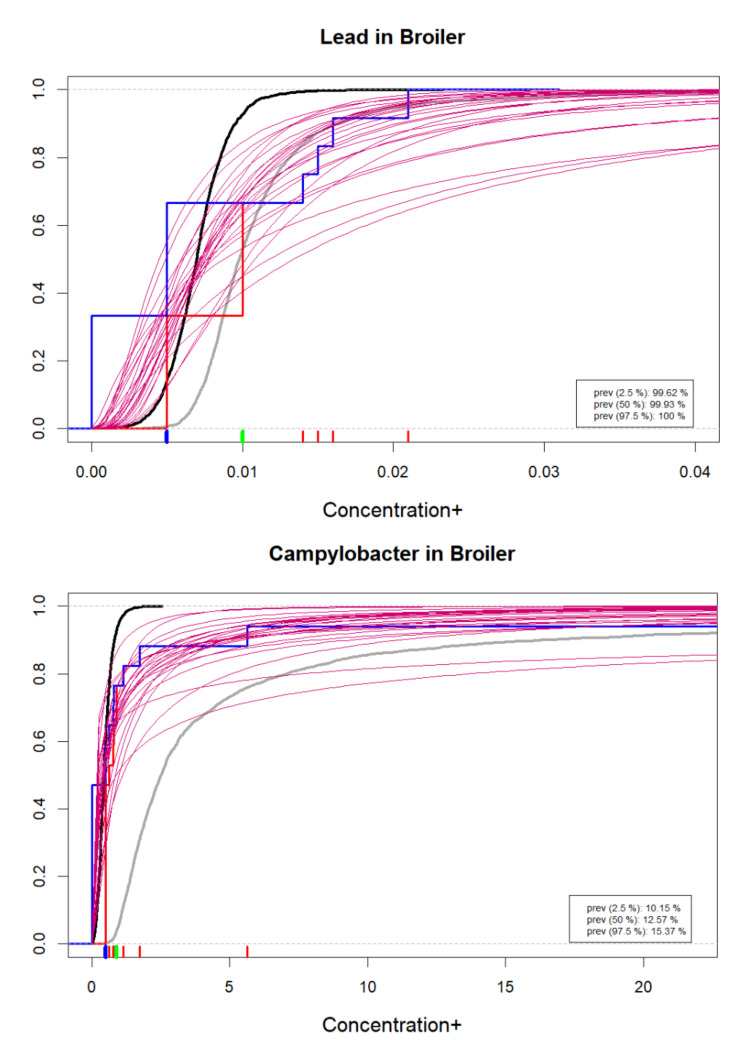
Example cumulative distributions of chemical (**top**) and microbiological (**bottom**) positive concentrations in the servings of the given food type. A sample of probable concentration distributions (magenta) and the uncertainty distributions for concentration mean (gray) and median (black). Observed data empirical cumulative distribution in blue/red with lowerbound/upperbound substitution of censored measurements is for comparison. Individual data points shown as red ticks, with green marks for values below LOQ and blue marks for values below LOD. Hazard prevalence estimate is given numerically. Numerical values on horizontal axis depend on the measurement unit used in the user’s data, e.g., mg/kg, μg/kg, CFU/g or CFU/mL, etc.

**Figure 3:**
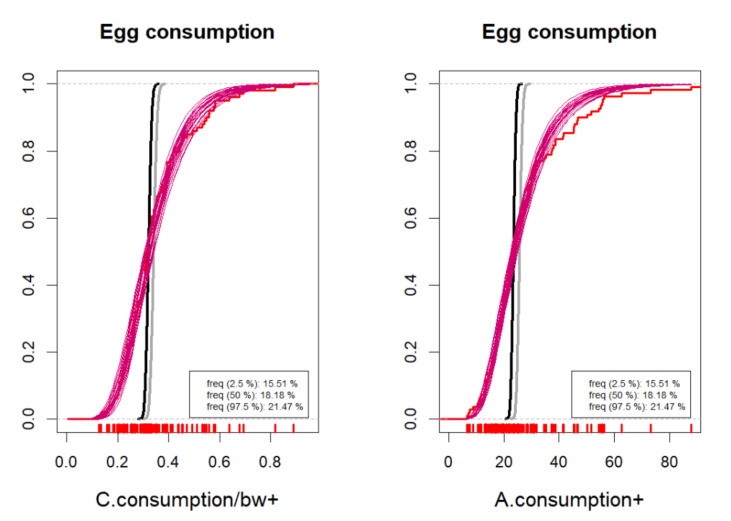
Example cumulative distributions of consumption on actual consumption days in the population of consumers. Chronic consumption per body weight (**left**) and acute absolute consumption (**right**). A sample of probable consumption distributions (magenta), and the uncertainty distributions for consumption mean (gray) and median (black). Observed data empirical cumulative distribution in red with data points shown as red ticks. Estimated frequency of consumption days is given numerically. Numerical values on horizontal axis depend on the measurement unit used in the user’s data, e.g., g, kg or mL etc.

**Figure 4:**
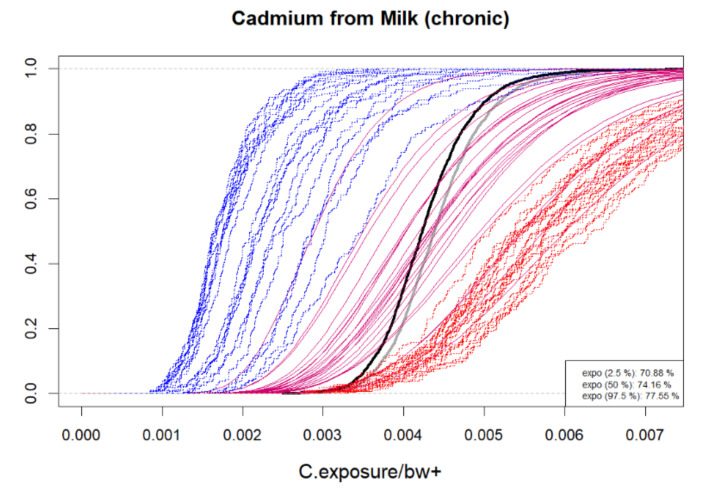
Example cumulative distributions of chronic exposure per body weight from contaminated food on consumption days. A sample of probable exposure distributions (magenta) and the uncertainty distributions for exposure mean (gray) and median (black). Monte Carlo pseudo empirical cumulative distributions produced for a few bootstrapped data sets with lowerbound/upperbound substitution of censored measurements (blue/red). Estimated exposure frequency is given numerically (i.e., proportion of days when contaminated food is consumed). Numerical values on horizontal axis depend on the measurement unit used in the user’s data.

**Figure 5:**
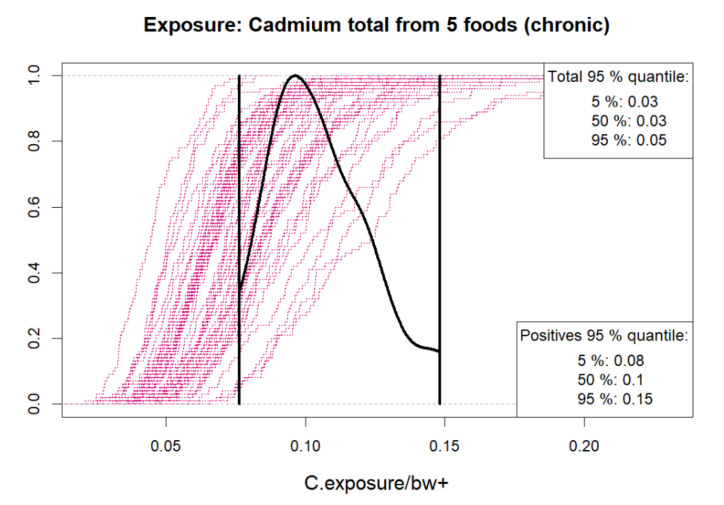
Uncertainty sample of cumulative distributions (magenta) of total positive exposures per body weight, from five selected food types, assuming positive concentrations & positive consumption days for all those foods. Uncertainty distribution of the chosen exposure quantile (here 95%) is shown as a density plot (black) within 90% uncertainty bounds (vertical bars). Numerical estimates for the chosen quantile are shown both for strictly positive exposure days only (lower corner) and for all days, which include zero consumption days and zero occurrences (upper corner). The “total quantile” hence represents the real exposure quantile whereas the “positives quantile” (and the plot) ignores zero exposure days. Numerical values on horizontal axis depend on what measurement units were used in the user’s data.

**Figure 6:**
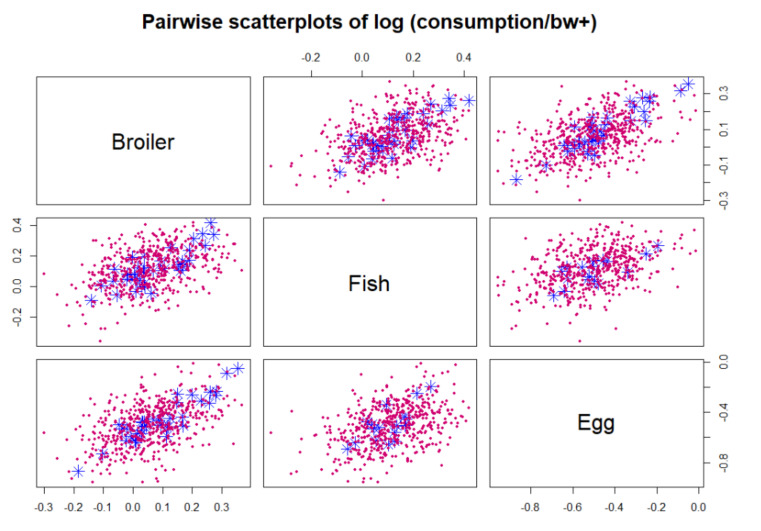
Example of pairwise correlations between log-consumption amounts on actual consumption days. Data points in blue and model simulated points in magenta (dots). Similar scatter plot is also available for mean log-consumption. Numerical values depend on the measurement units used in the user’s data, e.g., logarithm of kg or g per body weight.

The user can select between density plots and cumulative probability plots, either for absolute values or logarithmic values. These can be produced for one or more hazards, food types and result types according to selection, as in Figure 1. Since the distributions often tend to have long upper tails, particularly for concentrations, the density plots may not always be visually appealing. Cumulative distributions functions are therefore recommendable as shown in Figure 2, Figure 3, Figure 4 and Figure 5. As a default, the plots show distributions of concentrations, consumption amounts and exposures for positive values, excluding zeros. For some hazards and foods, the majority of the concentration values and/or consumption amounts may be zero or nearly zero, which would make their distributions difficult to plot due to a large peak at zero and a long thin upper tail. Therefore all plots show distributions for positives only. The proportion of positive concentrations or consumption days is then separately given as estimated in a legend box within the figure. Total exposure including all zero incidents (due to zero occurrences of a hazard or zero consumption days of a food, or both) is likewise given numerically. The exposure distributions can be inspected for single hazard-food pairs, or for the summed result from a subset of foods among all the specified foods in the model. All food types are nevertheless used for computing the full model to account for all their pairwise correlations. Pairwise correlations can be observed in a scatter plot for selected pairs of food types, as in Figure 6.

#### 6.1.4. Validation against Data

Data are not only used for estimations in BIKE, but also for comparisons between model and data. This provides a visual validation of sensible model fit against data. Empirical cumulative distributions are drawn from data together with model-based cumulative distributions. When concentration data contain censored values, two empirical cumulative distributions can be drawn to show the extremes: one based on lower bound substitution and the other based on upper bound substitution of the censored values. Comparisons to data apply directly to hazard concentrations and food consumption amounts. Concerning the exposure, there can be no observed data of the actual exposures of individuals that could be used for empirical data distributions since the occurrence data and consumption data are strictly two separate data sources. However, BIKE uses the basic non-parametric simulation approach for independently sampling from occurrence data and consumption data, then multiplying the random values, and plotting this ‘pseudo-empirical’ cumulative distribution of exposures. Uncertainty is visualized by plotting the same from several bootstrapped data. In this way, BIKE automatically shows comparisons between model-based distributions and non-parametric empirical distributions for model assessment and validation.

#### 6.1.5. Inspecting Uncertainty and Variability

The novelty throughout is quantification of both uncertainty and variability in the full model. All the plots for hazard concentrations and food consumption depict variability distributions. This may be for serving to serving variation of hazard concentration, variation of consumption amounts on random consumption days, or variation of average consumption between consumers. Likewise for acute and chronic exposures in the population of consumers. Each of these variability distributions are uncertain due to their uncertain parameters, and this uncertainty is depicted by visualizing a few probable distribution functions overlaid in the figure. The uncertainty is produced from the MCMC simulated posterior distribution of the model parameters. The uncertainty distribution of the mean and median are also depicted where applicable. Thus, uncertainty and variability are separated. In addition, the posterior predictive distribution (described in methods section above) is provided in tabular format.

#### 6.1.6. Adjustment Factors

All exposure distributions can be affected by applying constant processing factors to each food-hazard combination. Default values equal to one. The factor is assumed to affect the concentration levels, thus allowing to express expected differences in overall contamination level between the point of processing that is represented by the measurement data, and the actual point of consumption. For example, microbiological concentration data may represent raw meat, and this would need to be adjusted by a factor for cooked meat. Likewise, effects of washing, peeling and cooking could be accounted as with chemical hazards [40]. A constant factor acts as multiplication in absolute scale and addition in log-scale for concentrations. Similarly, for prevalence of hazards in foods, a factor between zero and one is applicable to reduce the estimated prevalence when simulating final exposures, e.g., after prevalence reducing management actions. These factors could also be used in scenario calculations as specified assumptions. There is no uncertainty distribution for these factors, since that would require highly context dependent models of cooking and storage.

## 7. Discussion

BIKE provides a user-friendly Bayesian parametric modeling approach that can be used for dietary exposure estimation and probabilistic uncertainty analysis with typical data sets, both microbiological and chemical. However, considering the vast variation in data quality and quantity, and structural differences between practical problems, no single dietary exposure assessment model can be expected to be perfectly fit for purpose in every imaginable situation. Yet, there is scope for a generic Bayesian inference tool. Any model is not to be applied in an automated manner to data without judgements of model performance with the data at hand. Visual comparisons between model distributions and empirical data are provided in BIKE to aid such judgement.

### 7.1. The Realm of Modeling in Dietary Exposure Assessment

Computation of a large Bayesian model with MCMC is a heavy task, even 20+ years [41] after the upsurge of general software such as WinBUGS/OpenBUGS. In practice, it may not be feasible to model simultaneously a very large number of hazards and foods, or very large data sets with BIKE. Therefore, it is best applicable in a tiered approach where the foods and hazards to be modeled are a preselected subgroup of interest, provided with reasonable data. At least minimal, but sufficient consumption data and occurrence data should be available for the food-hazard combinations to be assessed. For the users, that requires cautious judgement - just as with any statistical method. For BIKE, even a large proportion of concentrations can be allowed to be below detection limits LOD, or between LOD and LOQ, as long as a few measurements are above LOQ. Zero-inflated models can be used adjunct with the commonly reported concentration data format, to accommodate possible true zeros among censored values below LOD. Alternatively, all the concentration data can be taken to represent non-zeros accompanied by a separate sample data for prevalence estimation. Data may contain any combination of censored values with LOD and LOQ limits.

Currently, BIKE is only a dietary exposure model, relying on data to represent concentration levels thought to be relevant for dietary exposure. There are no inbuilt predictive models for bacteria growth or inactivation, nor cross contamination or process models for chemical or microbiological changes during food storage or preparation. Such models are highly context dependent for each hazard-food combination and might be added in further development. However, the user may apply simple adjustment factors for concentrations and prevalences to represent meaningful changes, e.g., to translate raw product concentration or prevalence to ready-to-eat concentration or prevalence, or to simulate a hypothetical scenario or an intervention.

In general, the consumption data collected over a short monitoring period (e.g., two days) may be limited about rare food types so that there would be little information about their consumption distributions. With smaller data samples, be it concentration or consumption, the uncertainties obviously become larger, as given by the Bayesian theory. Even when technically computable, in such case the assessment may not be enough accurate for confident decision-making, but it is still important to display the magnitude of uncertainty realistically. No model can provide information out of nothing, but one can strive to provide the best out of available data. Data sets with limited quantity and heterogeneous quality of information can be used in BIKE and their uncertainties assessed in a comparable way, making a probabilistically coherent synthesis. Data with various structures could be added to the model by coding the corresponding models likewise to those already shown in the open code. This would allow flexible extensions to the basic model, tailored to the available data. However, as with any Bayesian analysis using MCMC, it is the users’ responsibility to assess whether the simulations have properly converged, which may not be the case with over-parameterized models, unless deliberately informative prior distributions are placed. Although BIKE is not structurally over-parameterized, i.e., there is no inherent structural non-identifiability [42], small data can still lead to poor convergence, e.g., with censored data and zero-inflated models. This could leave parameter estimates very uncertain, albeit still computable from sufficiently long simulation runs.

### 7.2. Approaches to Uncertainty

Alternative non-Bayesian approaches to uncertainty quantification in dietary exposure assessment often rely on bootstrapping methods [43] or frequentist ‘distribution estimators’ [44,45]. Since some data sets can be quite small, non-parametric bootstrapping fails to fully quantify uncertainty [8] and parametric bootstrapping would depend on parameter estimates that are themselves likewise unstable. Frequentist ’distribution estimators’ a.k.a. fiducial or confidence distributions may be applicable to some individual parameters. For example, in the case of $\mu \;\&\;\sigma^{2}$ for normal distribution, these fiducial distributions are effectively the same (although with different interpretation of probability) as posterior distributions based on uninformative improper prior density $f\left(\mu ,\sigma^{2}\right)\propto 1/\sigma^{2}$. Such prior leads to nearly improper posterior distribution when data sample is very small, so the same concerns fiducial distributions. Fiducial distributions for $\mu \;\&\;\sigma^{2}$ are sometimes used in the manner of Bayesian distributions without noting the difference and the similarity of implicit improper prior. To ensure proper posterior distributions, default proper priors were used for model parameters in BIKE. However, other priors could always be coded instead. Probability models for complicated data patterns with multiple parameters would not provide pivotal quantities for fiducial solutions more generally. Then, different non-Bayesian methods would need to be tailored and combined for each case, in several steps, ad hoc. In contrast, the Bayesian approach is a consistent one-step method of probabilistic inference for multiple parameters, a general principle adopted in BIKE.

### 7.3. Parametric or Non-Parametric?

Combinations of parametric models can provide advantages over non-parametric methods for efficient uncertainty quantification [7], although they obviously depend on the chosen distribution models, which can constrain distribution shapes e.g., as with tail probabilities. This makes model choice another level of uncertainty, but this can be addressed by model comparisons. Currently, BIKE exploits log-normal distributions for their general usefulness and the distributions are visually compared against non-parametric data sample distributions. Log-normals are useful when modeling products of two or more variables, such as concentration and consumption amount whose product equals exposure, since the product of two log-normal variables follows another log-normal distribution that can be solved. The sum over several exposures has no similar solution, and needs to be simulated for an approximation. Multivariate normal distributions provide efficient ways for modeling parametric correlations between quantities, e.g., food types, for which this could be of interest. in BIKE, multivariate normal distributions were exploited for one-day food consumption amounts, mean consumption amounts, and consumption frequencies. It is not always clear beforehand if these correlations will be important features in a specific data set, but other correlation structures could be proposed as a further research.

When measurement data exhibit very large variance, the mean of log-normal distribution, $\exp(\mu +0.5\sigma^{2})$, also becomes large. Combined with large uncertainty of both μ and σ this may be less useful summary than median exp(μ). This occurs typically when a few concentration values are extremely large while most are small. For some data sets, gamma-distribution may provide better fit than log-normal, but some other useful properties would be compromised. With large sample size, the empirical distribution of data may become the best model of itself, but does not provide the analytical benefit that comes with parametric models. Combinations of parametric models provide many options for efficient uncertainty quantification and estimation, including possible extensions to common regression models and larger Bayesian hierarchical models for evidence synthesis from diverse data sets.

### 7.4. Advantages in Multivariate Multiparameter Exposure Assessments

Apart from small data problems, both challenges and opportunities come with complicated data patterns when making evidence synthesis from multiple data sets, concerning both consumption data and occurrence data in various formats. In general, parameter estimates from a single data set may not always be unique, as required by maximum likelihood estimation. They may be nearly non-identifiable (as on nearly flat ridges of likelihood), or even structurally non-identifiable from a single data set alone. The multiparameter uncertainties can be correlated, e.g., with mixture distributions where different parameter combinations may explain the data equally well. With more structured probability models, a patchwork of unrelated statistical methods for all uncertain parameters would not provide a consistent assessment of the joint uncertainty distribution. The advantage of Bayesian statistical models is by far their flexibility for extensions and coherent modeling of the uncertainty of all connected parameters jointly. This becomes increasingly needed when combining evidence from diverse data for multiparameter inference. That is, when parameters could not be estimated properly from one data set but by combining information from two or more data sets. An exposure estimation (as BIKE) may then be further integrated to risk estimation as e.g., for microbiological criteria [46], or ranking of risk management interventions. The main obstacle remains computational, a cost that can be well spent for a generally applicable method in food safety risk modeling. The need for specialized programming skills for self made MCMC algorithms can be avoided by using some of the existing general Bayesian tools, which then allow risk assessors to focus on model definitions rather than tedious engineering of MCMC samplers for each situation. However, an important prerequisite for still wider applicability is the availability of user interfaces providing some selection of models and analysis features as a tool. The BIKE interface is intended to provide this, allowing also new extensions to be developed transparently with open-source code.

### 7.5. Further Issues

It remains to apply BIKE with other and larger data sets, with suitable modeling options. New correlation structures may need to be developed for better scalability, as well as possible hierarchical model extensions for nested data structures and unbalanced data.

### 7.6. Obtaining BIKE

BIKE is found in github repository: https://github.com/jukran/BIKE (accessed on 22 September 2021).

## Abbreviations

The following abbreviations are used in this manuscript:

bw	body weight.
E(·)	expected value of ·.
f	probability density function.
F	cumulative probability function.
L	likelihood function, i.e., conditional probability of data.
LOD	Limit of detection.
LOQ	Limit of quantification.
LN	Log-normal distribution.
MCMC	Markov chain Monte Carlo simulation method.
MN	Multinormal distribution.

## Appendix A. Synthetic Data

Synthetic random data were generated in Excel for demo runs. These data only mimic real data and are loosely based on analysing various published summaries. Real original data would be subject to ownership rights. Therefore, these synthetic data were generated for free use. New random data sets of different sample sizes can then be generated by re-evaluating the random number functions in a downloadable Excel file.

From Finriski 2012 report [47] (part 2, Data Tables), the categorical weight distribution for men at 20 years of age (recalled at age 25–34) was simulated (uniformly within category, categories sampled as their relative proportions) and the result fitted as a log-normal distribution LN($\mu_{w}=$ 4.31, $\sigma_{w}=$ 0.14) for generating random body weights for the test data. The original weight categories and their proportions were [50–60, 60–70, 70–80, 80–90, 90–100, 100–110] kg and [5, 24.6, 36.7, 24.8, 4.1, 2.3] %. From EFSA Comprehensive European Food Consumption Database, acute consumption statistics on 5 food types (Foodex 2, levels L2 & L3) for consumption days were extracted for Finnish adults (study: Findiet 2012). Based on the reported means and standard deviations in absolute scale, parameters for corresponding log-normal distributions were obtained. The reported standard deviation was first reduced by a factor of 0.6 for not exceeding the upper quantiles excessively when simulating synthetic data. Since the EFSA database only provides summaries for one-dimensional distributions per each food type instead of raw data for investigating the multidimensional distribution, it is not possible to do sophisticated parameter estimation for re-creating similar but synthetic data. Also, the correlations between consumed food types are lost. However, simple synthetic correlations were crafted by setting a correlation coefficient 0.75 for each pair of log-body-weight and log-amount of consumed food type so that higher weights tend to combine with higher consumption, which also induces correlations between all food types. This is obviously not fully realistic, but suitable for demonstration purpose. The consumption amounts were thus generated from

$$P\left(\log x|\log w\right)=N(\mu_{x}+\frac{\sigma_{x}}{\sigma_{w}}\rho \left(\log w-\mu_{w}\right),\left(1-\rho^{2}\right)\sigma_{x}^{2})$$

where $\mu_{x},\sigma_{x}$ are parameters for the food consumption log-amounts in question, $\mu_{w}=4.31$, $\sigma_{w}=0.14$ for the consumer body log-weights and ρ=0.75 for the synthetic correlation.

The food categories (levels L2 or L3) were: unprocessed whole eggs (L3), fish (meat) (L2), birds meat (L3), milk (L3), leafy vegetables (L2). Accordingly, the consumption frequencies and marginal univariate log-normal distributions were 22% LN(3.20, 0.67), 23.3% LN(4.58, 0.48), 30.3% LN(4.49, 0.48), 76.6% LN(5.69, 0.53), 51.4% LN(3.60, 0.63).

Microbiological prevalence and concentration can vary hugely between the target food groups. Therefore, the following distributions used in the example data set mostly do not represent exactly matching food groups in the food consumption data and should be used only as examples of synthetic data for trying out the model. Most of these distributions do not describe final concentrations at the time of consumption without further adjustments. Hence, more representative data would be needed to assess any specific food product more realistically. Due to rare data representing concentrations at the actual time of consumption, microbiological concentrations in risk assessments are often merely predicted (based on earlier steps in the food chain) rather than estimated from models. Here, synthetic data for the concentrations was generated from log-normal distributions, by first roughly estimating the parameters μ,σ as follows. Prevalence *p* of contamination was either estimated together with these parameters using zero-inflated model, or separately from binomial sample data.

**Salmonella in broiler meat:** in [48], concentrations in random broiler filets (raw meat) at retail were reported. 201 observations were below detection limit, 19 were above with a stated estimate of cfu/filet. The weight of a filet was approximately 182 g, which was used for converting the distribution as per gram. Since observations below detection limit could be from both truly uncontaminated and truly contaminated filets, the data was re-analysed with a zero-inflated log-normal model with flat priors for μ and σ but vaguely informative Beta(2,4) prior for *p*. Posterior means of these parameters were (−6.5, 4.1, 0.45).

**Salmonella in milk:** the sample prevalence of Salmonella in milk and cream was reported in [49] as 4/3725=0.1%. Also, the prevalence range of 0–11.8% has been reported [50]. Concentration data from milk are rare, but in [51], nine positive concentration values (means of two measurements) from home-made ice cream were reported although these originated from raw eggs. With flat priors for μ and σ, posterior means of these were (5.399, 2.368). In comparison, concentration distribution in cattle faeces was estimated as LN(0.75 × log(10), 1.39 × log(10)) as an origin of contamination for raw milk in [50].

**Salmonella in eggs:** in the EU, sample prevalence of positive eggs 29/9700 was reported in [52]. In [53], 20 positive concentrations below 20 cfu/egg were reported (i.e., 1–20 cfu/egg) for raw eggs. Three concentrations were interval censored between 101–1000 cfu/egg and two were $1.5\times 10^{4}$ and $1.2\times 10^{5}$. Assuming egg weight 64 g, the distribution was converted as per gram. With flat priors for μ and σ, posterior means were used for parameter estimates leading to (−1.347, 2.992).

**Salmonella in fish:** the sample prevalence in raw fish was reported in [49] as 6/2086. Concentration data are very rare, but two out of three PCR positive samples (25 g) from black tiger prawns were reported in [54] as 40/100 cfu/g and <30/100 cfu/g. Taking these as 40/100, 1/25–30/100, and 1/100–1/25 and using flat priors for μ and σ, gives posterior distributions with fairly long tails. Therefore, posterior medians were used for parameter estimates (−2.318, 3.025).

**Salmonella in vegetables:** the sample prevalence of 6/1860 was reported in [55], and concentrations 0.02, 0.019, 0.019, 0.024, 0.024, 0.281 and >0.281 cfu/g were reported from various vegetables. With flat priors for μ and σ, the resulting posterior means for parameters were (−2.936, 2.152).

**Campylobacter in broiler meat:** prevalence and concentrations in retail raw broiler meat were described in [56]. Sample prevalence was 76/608, and 34 measurements were taken as exact and 42 as censored between 1/25–0.5. With flat priors for μ and σ this gives posterior means of parameters (−0.7075, 1.521).

**Campylobacter in milk:** in the EU, sample prevalence of positive milk units 30/1554 (1.9%) was reported in [52]. In a contaminated farm [57], concentrations 1, 2, 4, 5, 100 cfu/100 mL were reported and 6 were below detection limit, from samples of 500 mL. Assuming all 500 mL samples were contaminated, and detection limit of analysed samples was 1 cfu/100 mL, the 6 results were taken as censored within [1/500, 1/100] cfu/g. With flat priors for μ and σ this gives posterior means of parameters (−4.243, 1.97).

**Campylobacter in eggs:** in [58], sample prevalence of 11/2710 was reported (egg shells). Concentrations 240, 5×<3, 7.4, 3, 3×<3, 93, 15, 3.5, <3, 3, 2×<3 per 100 mL in positive egg yolks were reported in [59]. Assuming lower limits 1 per 100 mL for the left-censored positive results, and flat priors, the posterior means of parameters were (−3.237, 1.599).

**Campylobacter in fish:** Campylobacter in fish is generally rare [60]. In [61], Campylobacter jejuni was isolated in 36/240 samples of blue crab meat (50 g), but all quantitative levels were <0.3 MPN/g. Interpreting these as 36 values between [1/50, 0.3], and the rest as either true zeros or values <1/50, with flat priors on μ,σ and vague informative prior on *p* (Beta(2,4)) in a zero inflated model gives posterior means (−3.672, 0.706, 0.3123).

**Campylobacter in vegetables:** in [55], the sample prevalence of 3/1810 was reported, and concentrations 0.024, 0.024, 0.096 cfu/g. From only three measurements and flat priors, posterior distribution of μ and σ was computed. Since the posterior density of σ had very long tail, posterior mean of μ and posterior median of σ were used (−3.228, 1.671).

**Lead:** the chemical concentrations of lead in chicken meat, liquid milk, fresh eggs, fish meat and leaf vegetables were reported in [62]. Unfortunately no raw data were shown, but instead summaries obtained after substituting left-censored values with e.g., Middle Bound values. Also the reporting limits varied over the original measurements and were not shown in detail. Although proper parameter estimates for concentration distributions are not feasible from this information, rough estimates may be sketched. Using the mean and 95% percentile (Middle Bound substitutions) gives a standard deviation, and transforming these parameters to log-normal parameters gives distributions: broiler: LN(1.710, 1.244), milk: LN(0.719, 1.155), eggs: LN(1.575, 1.283), fish: LN(2.455, 1.128) vegetables: LN(3.422, 0.764), in μg/kg. Final synthetic data was simulated as μg/g with LOD = 0.005 and LOQ = 0.01 so that some values will be censored.

**Cadmium:** the chemical concentrations of cadmium in chicken meat, liquid milk, fresh eggs, fish meat and leaf vegetables were reported in [63]. Unfortunately, no raw data were shown, but instead only mean values obtained after substituting left-censored values with e.g., Middle Bound values. No information was shown on concentration variances or quantiles. The following means (μg/kg) from Middle Bound substitutions were reported: poultry 7.99, liquid milk 1.05, fresh eggs 3.33, fish meat 26.0, leaf vegetables 36.4. Assuming that the standard deviations are equal to means, and transforming to log-normal parameters gives distributions: broiler: LN(1.732, 0.833), milk: LN(−0.298, 0.833), eggs: LN(0.856, 0.833), fish: LN(2.912, 0.833), vegetables: LN(3.248, 0.833). Final synthetic data was simulated as μg/g with LOD = 0.001 and LOQ = 0.005, except LOQ = 0.002 for milk so that some values will be censored.

In all cases, real or synthetic data should contain at least some concentration values measured above LOQ (as ‘exact’) for the density estimation to be possible in BIKE. This may not automatically occur in small samples, so the data generator should be used cautiously. Likewise, real data should be inspected for a sanity check before modeling to assess whether there is sufficient information at all for the attempted estimation.
